# Effects of long-term fertilization on soil humic acid composition and structure in Black Soil

**DOI:** 10.1371/journal.pone.0186918

**Published:** 2017-11-02

**Authors:** Jiuming Zhang, Jingkuan Wang, Tingting An, Dan Wei, Fengqin Chi, Baoku Zhou

**Affiliations:** 1 College of Land and Environment, Shenyang Agricultural University, Shenyang, China; 2 Soil Fertilizer and Environment Resources Institute, Heilongjiang Academy of Agricultural Sciences, Key Laboratory of Soil Environment and Plant Nutrition of Harbin, Heilongjiang Province, China; RMIT University, AUSTRALIA

## Abstract

The composition and structure of humic acid (HA) can be affected by fertilization, but the short-term effects are difficult to detect using traditional analysis methods. Using a 35-year long-term experiment in Black Soil, the molecular structure of HA was analyzed with Fourier transform infrared spectroscopy (FTIR), ^13^C nuclear magnetic resonance spectroscopy (NMR), and fluorescence spectroscopy. Variation in HA was analyzed after long-term fertilization, including fertilization with manure (M), inorganic N, P and K fertilizer (NPK), manure combined with inorganic N, P, and K fertilizer (MNPK), and a no-fertilizer control (CK). The application of each fertilizer treatment increased crop yields compared with the CK treatment, and the MNPK treatment increased crop yield the most. The ratio of main IR absorption peak of HA at 2,920 cm^−1^ compared with the peak at 2,850 cm^−1^ (2920/2850) was higher in the NPK and MNPK treatments compared with the CK treatment. The application of manure (MNPK and M treatments) increased the ratio of hydrogen to carbon (H/C) in HA, and raised the ratio of the main IR absorption peak of HA at 2920 cm^−1^ to that at 1720 cm^−1^ (2920/1720). Manure treatments also raised the ratio of aliphatic carbon (C) to aromatic C, alkyl C to alkoxy C and hydrophobic C to hydrophilic C and the fluorescence index (f 450/500), but decreased the degree of aromatization of HA, when compared with the CK treatment. The ratio between each type of C in HA was similar among all the fertilizer treatments, but NPK had a lower ratio of H/C and a lower content of aliphatic C compared with the CK treatment. These results indicated that the molecular structure of HA in Black Soil tends to be aliphatic, simpler, and younger after the application of manure. While the application of inorganic fertilizers increased in the degree of condensation of HA and made HA structure complicated. The application of manure alone or combined with inorganic fertilizers may be an effective way to increase crop yield and improve the structure of soil organic matter.

## Introduction

Organic matter plays important roles in soil fertility, environmental protection, and sustainable agricultural development. Humus, as the main component of soil organic matter, affects a variety of soil properties and forms. The quantity, composition and nature of humus reflect certain conditions and processes of pedogenesis, and these factors are important indicators of soil fertility [1]. Humic acid (HA) is an important component of soil humus, and its composition, structure and properties are directly related to soil fertility and crop yield [2]. The addition of organic materials increases the content of HA in soil and changed its chemical structure [3–4]. Galantini and Rosell [5] found that fertilized soil raises the contents of aliphatic and phenolic hydroxyl groups in HA compared with unfertilized soil. Although other research found that the fertilization has little changes on the elemental composition of HA and increases the relative molecular mass [6].

Humic substances are mainly composed of C, H, O, N, P, S, and a small amount of Ca, Mg, Fe, Si and other ash elements. In the past decades, the methods for analyzing HA structure have been relatively limited. Now modern analytical technologies, such as ^13^C nuclear magnetic resonance (^13^C-NMR), infrared (IR) spectroscopy, and fluorescence spectroscopy are used to analyze the structure of soil humus [7–10]. Wen et al. [11] determined the content and structure of humus components in peat soil and analyzed aliphaticity and polarity size by using organic chemistry and spectroscopy methods. Based on NMR and IR spectroscopy methods, Soumia et al. found a close relationship between oxidation processes and aromatization after the composting activated sludge and green manure [12]. However, little has been published on the changes in the structure and element composition of HA after long-term application of manure and inorganic fertilizers in Black Soil.

The present study employed an experimental station of arable land conservation and environmental monitoring (a 36-year Black Soil fertilization experiment) conducted by the Ministry of Agriculture in Heilongjiang Province, China. We used the methods of Fourier transform IR spectroscopy, ^13^C NRMS, and fluorescence spectroscopy to analyze the molecular structure of HA and discuss the effect of fertilization (especially manure and inorganic fertilizer) on the element composition and molecular structure of HA in Black Soil. The results of this research would provide a theoretical basis for the characteristic of HA structure. We hypothesized that the manure application would increase the element content in HA and make HA structure more aliphatic compared with soils given inorganic fertilizer and those receiving no-fertilizer application.

## Materials and methods

### Study site and soil sampling

The experiment was conducted at the experimental station of arable land conservation and environmental monitoring (45°50' 30''N, 126°51' 05''E) established by Ministry of Agriculture, Heilongjiang Province, China, in 1979. Ministry of Agriculture concerned with the protection and sustainable development of Black Soil resource. Different types of fertilizer and rotation system were required for sustainment and then improvement of the fertility and productivity in Black Soil for long-term. This field studies did not involve endangered or protected species. The soil in this region is classified as Black Soil (Luvic Phaeozem, FAO Classification), developed from a loess-like parent at 151 m elevation. The temperate continental monsoon climate has ≥10 annual average effective accumulated temperature of 2,700°C, the annual rainfall is 533 mm, and the frost-free period is 135 days. In 1979, the soil has 15.50 g kg^-1^ total C, 1.47 g kg^-1^ total nitrogen, 1.07 g kg^-1^ total phosphorus, 25.16 g kg^-1^ total potassium, 151.1 mg kg^-1^ alkali-hydrolysable nitrogen, 51.0 mg kg^-1^ available phosphorus, pH (H_2_O) 7.2, 16.7% sand, 58.4% silt, and 24.9% clay at 0–20 cm depth. A wheat-soybean-maize rotation system was adopted in this station from 1980.

Four fertilization treatments were selected in this station: (1) no-fertilizer control (CK); (2) inorganic N, P, and K fertilizer (NPK); (3) manure (M); (4) manure combined with inorganic N, P, and K fertilizer (MNPK). The amount of each fertilizer is shown in Table 1. Manure was horse compost and was applied at an amount of 75 kg N ha^-1^ (about 18,600 kg ha^-1^ horse manure) after maize was harvested. The contents of N, P (P_2_O_5_), and K (K_2_O) in manure were 5.8 g kg^-1^, 6.5 g kg^-1^ and 9.0 g kg^-1^, respectively. N fertilizer was urea (N 46%) and was applied in autumn at wheat and soybean rotation seasons, while half was applied at the 12-leaf stage and the other half was applied in autumn at maize rotation season. P fertilizer was triple superphosphate (P_2_O_5_ 46%), and K fertilizer was potassium sulfate (K_2_O 50%). They were all applied in autumn. Each plot was 36 m^2^ and there were four replicates of each plot type in the field. Soil samples were collected from 0–20 cm soil depth in 2012. The basic soil characteristics are shown in Table 2. Maize was harvested at the mature stage, and yield was calculated.

**Table 1:** Application amount of different fertilizers at wheat-soybean-maize rotation season.

Treatment	Wheat season			Soybean season			Maize season			
	Inorganic N fertilizer (kg N ha^−1^ y^−1^)	Inorganic P fertilizer (kg P_2_O_5_ ha^−1^ y^−1^)	Inorganic K fertilizer (kg K_2_O ha y^−1^)	Inorganic N fertilizer (kg N ha^−1^ y^−1^)	Inorganic P fertilizer (kg P_2_O_5_ ha^−1^ y^−1^)	Inorganic K fertilizer (kg K_2_O ha y^−1^)	Inorganic N fertilizer (kg N ha^−1^ y^−1^)	Inorganic P fertilizer (kg P_2_O_5_ ha^−1^ y^−1^)	Inorganic K fertilizer (kg K_2_O ha y^−1^)	Manure (t·ha^−1^ y^−1^)
CK	0	0	0	0	0	0	0	0	0	0
NPK	150	75	75	75	150	75	150	75	75	0
M	0	0	0	0	0	0	0	0	0	18.6
MNPK	150	75	75	75	150	75	150	75	75	18.6

CK, no-fertilizer control treatment; NPK, inorganic nitrogen (N), phosphate (P), and potassium (K) fertilizer treatment; M, manure treatment; MNPK, treatment of manure combined with inorganic N, P, and K fertilizers

**Table 2:** Basic properties of soil samples in different fertilization treatments of Black Soils in 2012.

Treatment	Soil organic carbon (g kg^-1^)	Carbon of humic acid (g kg^-1^)	Total nitrogen (g kg^-1^)	Total phosphorus (g kg^-1^)	Total potassium (g kg^-1^)	Available nitrogen (mg kg^-1^)	Available phosphorus (mg kg^-1^)	Available potassium (mg kg^-1^)	pH
CK	12.41±0.11c	2.88 ±0.08c	1.21±0.03c	0.68±0.02c	27.1±0.67c	103.9±2.04c	22.3± 0.69d	138.4±2.31c	7.1±0.12a
NPK	14.39±0.12b	2.95 ±0.09c	1.46±0.06b	1.42±0.11a	28.4±1.23c	130.4±2.37b	154.6±2.41a	165.4±2.23b	6.4±0.06c
M	14.50±0.20b	4.43 ±0.08b	1.51±0.02a	1.02±0.08b	30.3±1.36b	123.8±4.37b	142.0±5.32c	135.8±2.33c	7.2±0.15a
MNPK	14.79±0.18a	5.03 ±0.06a	1.54±0.02a	1.48±0.04a	31.5±1.32a	135.7±3.17a	147.8±2.41b	215.6±2.52a	6.9±0.04b

CK, no-fertilizer control treatment; NPK, inorganic nitrogen (N), phosphate (P), and potassium (K) fertilizers treatment; M, manure treatment; MNPK, treatment of manure combined with inorganic N, P, and K fertilizers.

Different lowercases in the same column indicate significant differences at *P*<0.05 between different fertilization treatments.

### Determination of soil characteristics and methods

Air-dried soil samples (2 mm) were weighed (about 100 g) in a glass bottles. Then, 1 mol L^−1^ HCl was added at a soil–water ratio of 1:1. The mixture was held at room temperature for 1h, then adjusted to a final soil–water ratio of 1:10 with 0.1 mol L^−1^ HCl. The bottle was then shaken for 1 h at room temperature. The mixture was centrifuged at low speed (3,500 r min^−1^), after which the supernatant was discarded. After centrifugation with 1 mol L^−1^ NaOH, the precipitate (pH = 7) was held for 1h. Then, 0.1 mol L^−1^ of NaOH was added to achieve a final soil–water ratio of 1:10. Each tube was then immediately filled with nitrogen (1 min) and sealed. The glass bottles were sealed under nitrogen (capping), after standing for 12 h overnight. The next day, low-speed centrifugation was used and the liquid in the bottle was acidified to pH 1.0 with 6 mol L^−1^ HCl, and held for 12–16 h. The extraction was repeated three times; after centrifugation at low speed, the precipitant was used as the HA content of the sample. This HA precipitant was then dissolved in a small amount of 0.1 mol L^−1^ KOH solution, after which solid KCl was added, so that the concentration reached K + 0.3 mol/L. After standing at room temperature for 1 h, high speed centrifugation (8,000 r min^−1^, 20 min) was employed and the supernatant retained. The supernatant was adjusted to pH 1.0 with 6 mol L^−1^ HCl and held for 12–16 h. After centrifugation, the supernatant was discarded. The HA was soaked in 0.1 mol L^−1^ HCl + 0.3 mol L^−1^ HF mixed acid, and then washed to an ash content of less than 1%. Next, the glass bottles were placed into an electrodialysis chamber for electro-osmosis, until AgNO_3_ in distilled water without Cl^−^ was observed. The liquid was concentrated by rotary evaporation and then freeze-dried for later use [13].

The elemental composition of HA was determined with an elemental analyzer (Elementar Analyser system GmbH, Hanau, Germany) by using the CHN mode. The content of O + S was calculated by the subtraction method, and the data from the elemental analysis were corrected using the ash and moisture data of the differential thermal analysis.

IR spectroscopy is used to determine one or several components in various substances based on the selective absorption of IR light by the organic functional groups (C-H, O-H, N-H, etc.) [14]. The attribution of absorption peak in IR spectrum is shown in Table 3. IR spectroscopy was performed using a Nicolet EZ360 IR spectrometer (Nicolet Instrument Corp., Madison, WI, USA), and a scanning mode of 4000–400 cm^−1^, using the KBr tablet method determination. The organic matter samples were vacuum freeze-dried and then pulverized to less than 2 μm. The soil organic matter samples and the KBr powder were then weighed using a micro- or semi-microbalance, as appropriate. Next, the sample–KBr ratio of 1: 200, agate in the mortar after the grinding compound, and the zero absorption point at 4 000, 2 000 and 860 cm ^−1^. The absorption intensity was measured by a three-point straight line as a baseline, and the results were compared. The resolution of the instrument was set to 4 cm^−1^.

**Table 3:** Attribution of each absorption peak of IR spectrum.

Absorption frequency (cm^-1^)	Vibration mode	Group
3407∼3384	O–H stretch	Phenolic compound, hydroxy group
2930∼2920	C–H stretch	Aliphatic compound
1720	C = O stretch	Carboxyl, aldehyde, ketone
1650∼1640	C = O stretch	Amino compound, carboxylate
1630∼1600	C = C stretch	Aromatic ring, alkene
1570∼1500	N–H OH deformation, C = N stretch	Amino compound
1515∼1510	Aromatic C = C stretch	Lignin
1460∼1450	C–H stretch	Aliphatic compound
1421∼1410	O–H OH deformation, C–O stretch	Aromatic oxide, phenol
1320	C–N stretch	First, second aromatic amine
1240∼1220	C–O stretch, OH deformation	Carboxyl
1080∼1030	C–O stretch	Polysaccharide, polysaccharide substance

^13^C-NMR spectroscopy is one of the most effective methods to qualitatively and quantitatively determine the structure of soil organic matter of undisturbed bulk soils [15]. A solid state NMR spectroscope (Swiss Bruker AV400-type NMR instrument, Bruker Instruments, Inc., Karlsruhe, Germany), using cross polarization magic angle spin (CPMAS) technology, was used for NMR analysis. The ^13^C resonance frequency was 400.18 MHz, the magic angle spin frequency was 8 kHz with a contact time of 2 ms, and the cycle delay time was 3 s. We collected 3,000 data points. The chemical shift was corrected with the external standard 2,2-dimethyl-2-silane-5-sulfonate. The integral area was given automatically by the instrument. The relative C content of each type of chemical shift interval integral area of the total integral area of the percentage was determined. The ^13^C NMR spectrum of humus can be divided into four major resonance regions as follows: 1) alkyl-C region (0 to 50 ppm), 2) alkoxyl-C region (50 to 110 ppm), 3) aromatic-C region (110 to 160 ppm) and 4) carbonyl-C region (160 to 200 ppm) [16]. In general, we observed absorption peaks of the main C-alkyl groups in the respective resonance regions and the attribution of the peaks at 21 ppm, 26 ppm, 30 ppm, 33 ppm and 44 ppm; these represented CH_3_, CH_2_, (CH_2_)n long chain C, crystal form (CH_2_)n long chain C and branched alkyl C [17], respectively.

The advantages of Fluorescence spectroscopy are that it has high sensitivity and good selectivity, and it does not destroy samples. Fluorescence spectrometry was performed using an LS50B luminescence spectrometer (Perkin-Elmer, Bucks, UK). The settings were as follows: excitation light source of a 150-W xenon arc lamp; PMT voltage, 700 V; SNR > 110; Bandpass, Ex = 10 nm; Em = 10 nm; response time, automatic; and scanning speed, 1,500 nm min^−1^. The spectrum was scanned for automatic instrument calibration. The sample scan concentration was 25 mg (C)/L. Data were collected using the software provided with the product, FL WinLab software (Perkin-Elmer). The 3-dimensional fluorescence spectra of the emission wavelength of Em covered 250–700 nm.

Soil pH was measured on a 1:2.5 (w/v) mixture of soil and water with a pH meter (Delta 320, Mettler Toledo, Switzerland). Soil total C and N concentrations were analyzed using an elemental analyzer (Vario EL III, Elementar, Germany). Because Black Soil in the study area is free of carbonate, soil total C accurately represents soil organic carbon (SOC). Total P and total K concentrations were determined by digesting samples in sodium hydroxide and then measuring, respectively, by ascorbic acid colorimetry and by atomic absorption [18].

### Statistical analysis

IR spectroscopy analysis was performed by using Nicolet Omnic 8.0 software (Thermo Nicolet Co., USA). Nuclear magnetic resonance spectroscopy (CPMAS^13^C-NMR) was determined by using MestReNova professional analysis software (Mestrelab Research SL, Santiago de Compostela, Spain). Fluorescence spectra were collected using FL WinLab software (Perkin-Elmer). After analyzing and extracting the source data, Microsoft Office Excel 2010 and Origin 8.0 software were used for data processing and preparing figures. The Origin plot was used to draw the data in the “Available Data” as a fit curve fit, and SPSS 19.0 statistical analysis software was used for the significant difference tests (Duncan method).

## Results

### Crop yield and humic acid content

**Fig 1:**
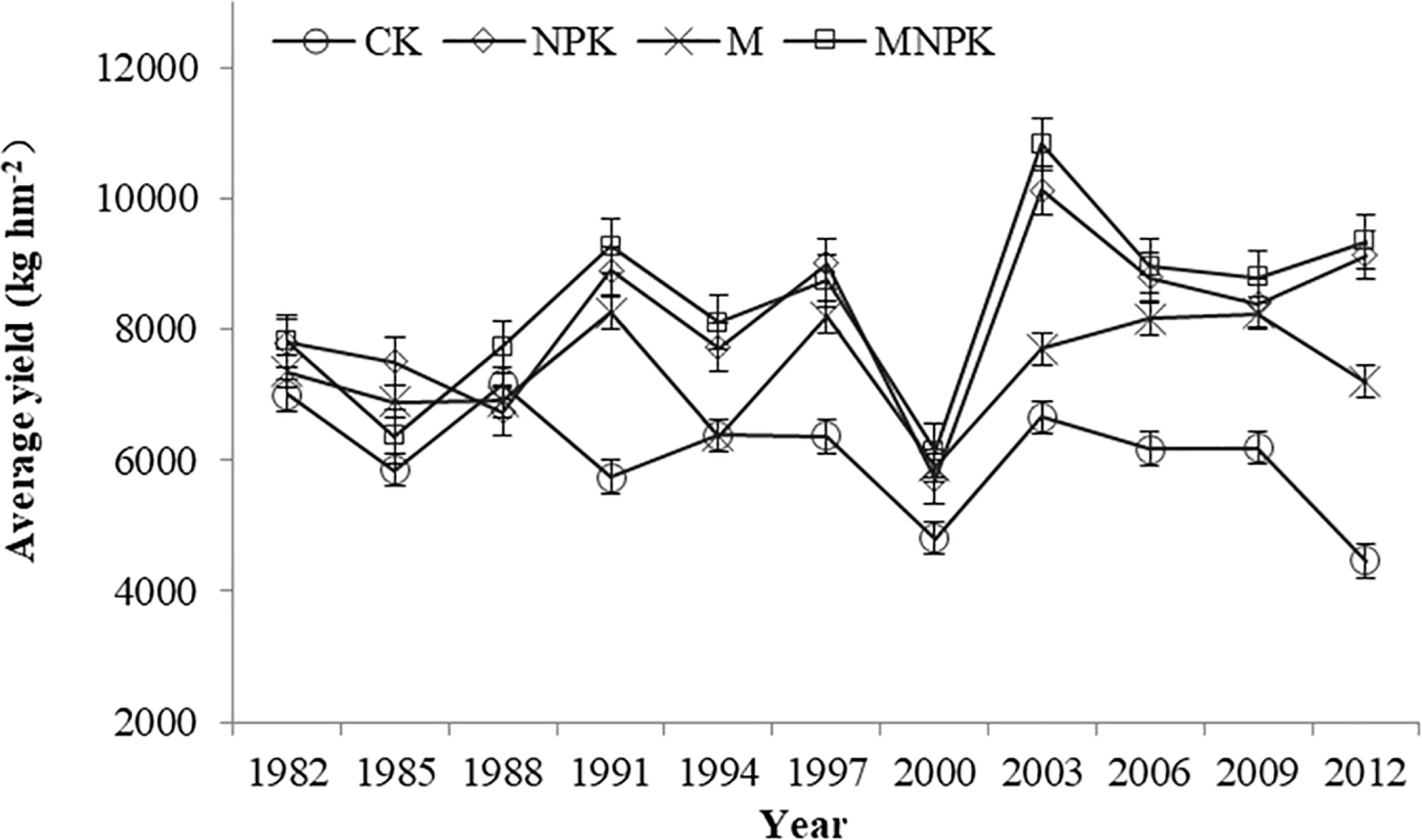
Crop yields by year in different fertilization treatments of Black Soil. CK, no-fertilizer control treatment; NPK, inorganic nitrogen (N), phosphate (P), and potassium (K) fertilizer treatment; M, manure treatment; MNPK, treatment of manure combined with inorganic N, P, and K fertilizers.

Long-term application of manure alone (M), or combined with inorganic fertilizers (MNPK) and inorganic fertilizers (NPK) increased the average yield of crop by 21.5%, 37.9% and 34.4%, respectively, compared with CK (Fig 1). The crop yield showed an increased trend with years (except in 2000, during which there was a severe drought).

The content of SOC in MNPK, M, and NPK treatments, respectively, increased by 19.2%, 16.8%, and 16.0% compared with the CK treatment (Table 2). Also, the C content of HA in soil was twice as high in the manure (MNPK and M) treatments as it was in the CK and NPK treatments (Table 2).

### Elemental composition of humic acid

The range of the content of various elements in soil HA was as follows: C content was 532.4 to 546.4 g kg^−1^, N content was 34.13–36.72 g kg^−1^, H content was 41.56–45.15 g kg^−1^, and O + S content was 377.9–388.0 g kg^−1^ (Table 4). The contents of N and H in HA were higher in manure treatments (M and MNPK) than in NPK and CK treatments. While M or NPK treatment did not significantly (*P*>0.05) increased the contents of C and (O + S) in HA. The H/C ratios of soil HA were increased by 5.3%, 5.2% and 4.3% in MNPK, M and NPK treatments when compared with the CK. The highest C/N ratio was found in NPK treatment, and the lowest in M and MNPK treatments. The O/C ratio was not significantly (*P*>0.05) affected by fertilization.

**Table 4:** Elementary composition of humic acid in different fertilization treatments of Black Soil.

Treatment	N (g kg^-1^)	C (g kg^-1^)	H (g kg^-1^)	(O+S) (g kg^-1^)	C/N	H/C	O/C
CK	34.59±0.37b	534.9±0.55a	42.52 ±1.39bc	388.0±4.26a	15.46±0.25ab	0.954±0.06b	0.544±0.05a
NPK	34.13±0.29b	546.4±0.92a	41.56±0.47c	377.9±3.52a	16.01±3.45a	0.913±0.18b	0.519±0.15a
M	36.30±0.17a	539.6±1.74a	45.15±4.13a	378.9±6.37a	14.87±0.37b	1.004±0.21a	0.527±0.07a
MNPK	36.72±0.55a	532.4±1.31a	44.57±1.25b	386.3±3.74a	14.50±3.54b	1.005±0.08a	0.544±0.09a

CK, no-fertilizer control treatment; NPK, inorganic nitrogen (N), phosphate (P), and potassium (K) fertilizers treatment; M, manure treatment; MNPK, treatment of manure combined with inorganic N, P, and K fertilizers.

Different lowercases in the same column indicate significant differences at *P*<0.05 between different fertilization treatments.

### Infrared spectroscopic analysis of humic acid

**Fig 2:**
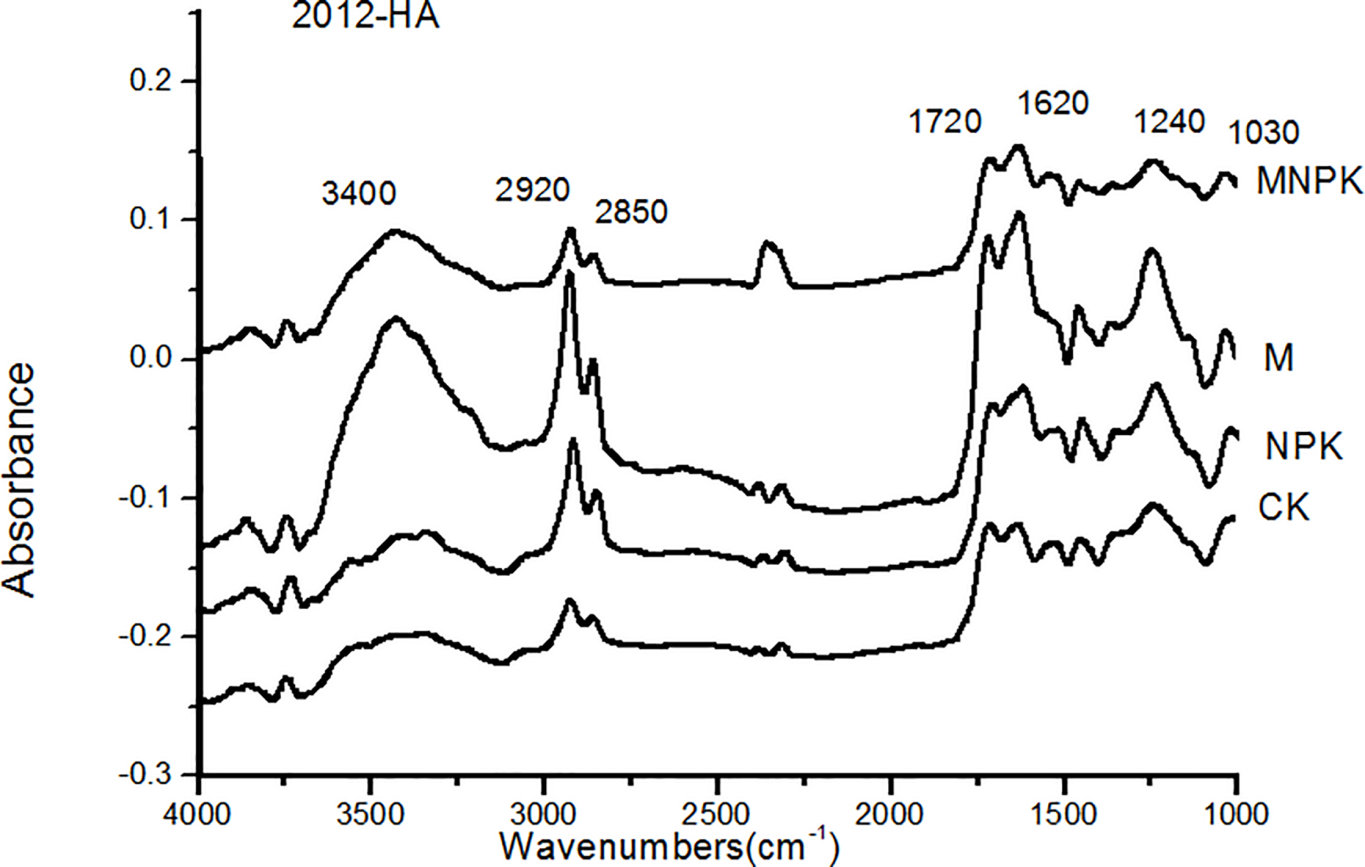
Infrared spectra of humic acid in 2012 (2012-HA) in different fertilization treatments of Black Soil. CK, no-fertilizer control treatment; NPK, inorganic fertilizers of nitrogen (N), phosphate (P), and potassium (K) fertilizer treatment; M, manure treatment; MNPK, treatment of manure combined with inorganic fertilizers of N, P, and K fertilizers.

The IR spectra of HA in Black Soil had similar characteristics, but the absorption intensity varied under different fertilization treatments (Fig 2). The absorption peak of IR spectrum in HA was shown at 3400, 2920, 2850, 1720, 1620, 1240, 1330 cm^-1^ and the strongest peak of stretching vibration was found at 2920 cm^−1^ (aliphatic polymethylene) and 1620 cm^−1^ (aromatic) in HA of Black Soil. MNPK and M treatments had the highest absorption peak at 3400 cm^−1^.

The ratios of IR absorption peaks at 2920 cm^−1^ to that at 1720 cm^−1^ (2920/1720) decreased in the order of M > MNPK > NPK > CK (Table 5). The ratios of IR absorption peaks at 2920 cm^−1^ to those at 1620 cm^−1^ (2920/1620) were the highest in the NPK treatment and the lowest in the M treatment. The MNPK and NPK treatments had a higher ratio of IR absorption peaks at 2920 cm^−1^ to that at 2850 cm^−1^ (2920/2850), whereas the M treatment had a lower 2920/2850 ratio compared with the CK treatment.

**Table 5:** Relative intensities of the main absorption peak of humic acid infrared spectra in different fertilization treatments of Black Soil (semi-quantity, cm^−1^).

Treatment	2920	2850	1720	1620	1240	1030	2920/1720	2920/1620	2920/2850
CK	0.905±0.01d	0.310±0.01c	1.112±0.01c	0.888±0.01c	1.093±0.01c	1.368 ±0.01c	2.919 ±0.10c	0.905±0.01d	0.310±0.01c
NPK	1.752±0.05c	0.402±0.01b	2.021±0.02b	1.295±0.01b	1.066±0.02c	1.663 ±0.06a	4.358 ±0.05b	1.752±0.05c	0.402±0.01b
M	2.059±0.04b	1.077±0.08a	2.304±0.05a	3.033±0.01a	1.361±0.08a	1.034 ±0.03d	1.912 ±0.14d	2.059±0.04b	1.077±0.08a
MNPK	2.180±0.03a	0.369±0.01bc	2.032±0.01b	1.622±0.03b	1.254±0.02b	1.572 ±0.03b	5.908 ±0.06a	2.180±0.03a	0.369±0.01bc

CK, no-fertilizer control treatment; NPK, inorganic nitrogen (N), phosphate (P), and potassium (K) fertilizers treatment; M, manure treatment; MNPK, treatment of manure combined with inorganic N, P, and K fertilizers.

Different lowercase in the same column indicate significant differences at P<0.05 between different fertilization treatments.

2920/1720, the ratio of the area of 2920 + 2850 to the area of 1720.

2920/1620, the ratio of the area of 2920 + 2850 to the area of 1620.

2920/2850, the ratio of the area of 2920 to the area of 2850.

### ^13^C-NMR spectral characteristics of HA

**Fig 3:**
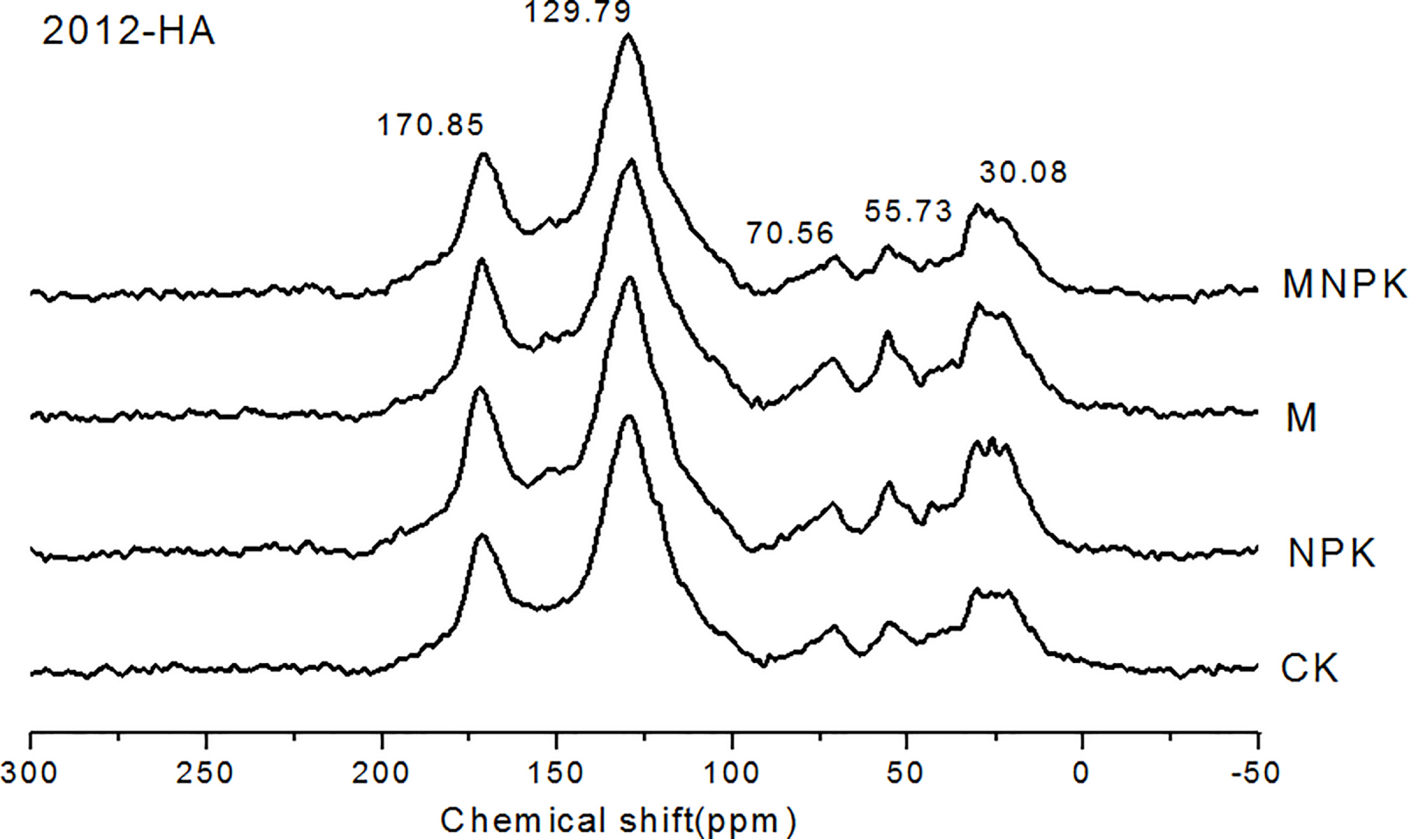
The results of solid-state polarization magic-spin ^13^C nuclear magnetic resonance spectroscopic analysis of humic acid in 2012 (2012-HA) in different fertilization treatments of Black Soil. CK, no-fertilizer control treatment; NPK, inorganic fertilizers of nitrogen (N), phosphate (P), and potassium (K) fertilizer treatment; M, manure treatment; MNPK, treatment of manure combined with inorganic fertilizers of N, P, and K fertilizers.

The ^13^C-NMR spectral absorption peak of alkyl-C in HA of Black Soil was mainly around 30 ppm, which represented the chemical shift of methylene C in long-chain alkane or cycloalkane structure (Fig 3). The absorption peak of alcoxyl C in HA was mainly at 55 ppm, which can be attributed to the absorption of methoxy C and carbohydrate C (Fig 3). In the aromatic C region, 129 ppm was mainly represented aromatic C substituted by carboxyl or carboxymethyl and with O, N, and other substituents at the position of the connection of H aromatic C absorption. In the carbonyl C region, the main signal appeared near 170 ppm, which represented the absorption of carboxylic acid, ester, and amide C [19].

The proportion of alkyl C in HA ranged from 19.1% to 21.8%, that of alkoxy C from 17.0% to 18.6%, that of aromatic C from 42.1% to 44.3%, that of carbonyl C from 17.8% to 19.9%, and that of aliphatic C (the sum of alkyl C and alkoxy C) from 37.4% to 39.7% (Table 6). The application of manure (M and MNPK treatments) and inorganic fertilizer (NPK) increased the content of alkyl C but decreased the content of aromatic C. The content of aliphatic C (the sum of alkyl C and alkoxy C) was the highest in M (39.7%), followed by MNPK (38.0%); it was lowest in CK and NPK (37.5%).

**Table 6:** C distributions of different types of humic acid in solid-state polarization magic-spin ^13^C nuclear magnetic resonance spectroscopic analysis in Black Soil under different fertilization treatments.

Treatment	Alkyl C (%)	Alkoxy C (%)	Aromatic C (%)	Carbonyl C (%)	Aliphatic C/Aromatic C	Alkyl C/ Alkoxy C	Hydrophobic C/Hydrophilic C
CK NPK M MNPK	19.1±0.15d	18.4±0.49a	44.3±0.45a	18.3±0.40b	0.84±0.02c	1.04±0.03c	1.73±0.02a
	20.4±0.29c	17.0±0.36b	42.9±0.92b	19.6±0.56a	0.87±0.03bc	1.20±0.03b	1.73±0.07a
	21.1±0.31b	18.6±0.55a	42.4±0.83b	17.8±0.38b	0.94±0.01a	1.13±0.05b	1.74±0.02a
	21.8±0.30a	16.2±0.53b	42.1±0.60b	19.9±0.50a	0.90±0.02ab	1.34±0.06a	1.77±0.05a

CK, no fertilizer treatment; NPK, inorganic fertilizers of nitrogen (N), phosphate (P), and potassium (K) treatment; M, manure treatment; MNPK, treatment of manure combined with inorganic fertilizers of N, P and K.

Different lowercase in the same column indicate significant differences at P<0.05 between different fertilization treatments. Aliphatic C/Aromatic C = (Alkyl C+ Alkoxy C)/Aromatic C.

Hydrophobic C/Hydrophilic C = (Alkyl C+ Aromatic C)/ Alkoxy C +Carbonyl C).

Compared with the CK treatment, the application with manure (MNPK and NPK treatments) significantly increased (*P*<0.05) the ratio of aliphatic C to aromatic C and that of alkyl C to alkoxy C in soil HA. The ratio of hydrophobic C to hydrophilic C in soil HA was not significantly different (*P*>0.05) among different treatments.

### Fluorescent 3-dimensional spectroscopic analysis of HA

**Fig 4:**
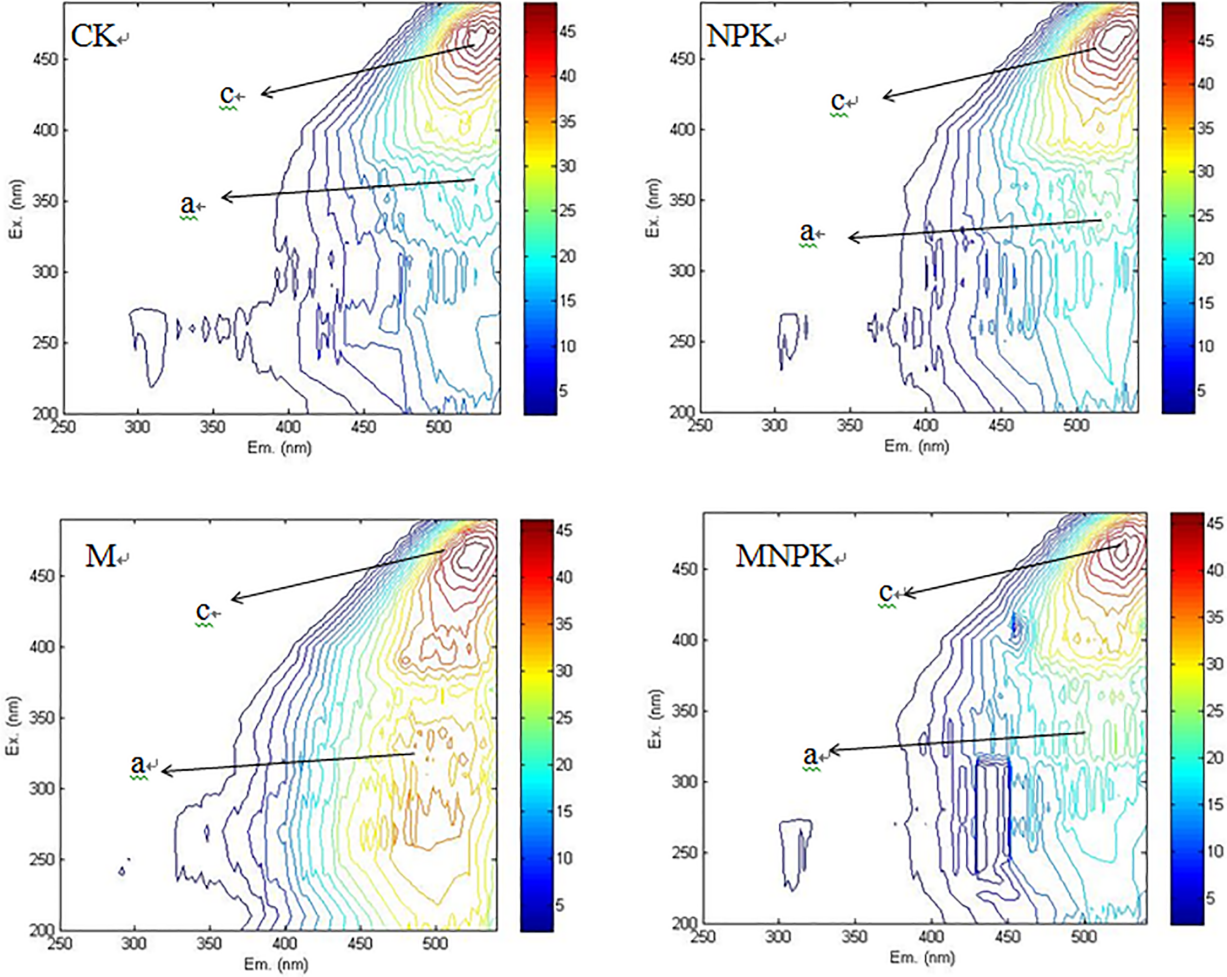
Three-dimensional excitation-emission matrices of humic acid under soil removal in four treatments: CK, no-fertilizer control treatment; NPK, inorganic nitrogen (N), phosphate (P), and potassium (K) treatment; M, manure fertilizer treatment; MNPK, treatment of manure combined with inorganic of N, P, and K fertilizers. Ex, excitation; Em, emissions. Peak A is the ultraviolet region type fulvic acid fluorescence peak; peak C is in the class of humic acid fluorescence peaks.

The 3-dimensional fluorescence spectrum can show the fluorescence characteristics of an analyte better than other methods because it can simultaneously obtain excitation and emission wavelengths of real-time change, and a 3-dimensional spectrum is superior to the traditional spectrum [17]. After the application of manure, the 370~450 nm wavelength in soil HA exhibited hypsochromic shift. Different fertilization treatments produced two characteristic peaks with the excitation to emissions ratios at 460–470 nm/518–530 nm and at 330–340 nm/478–520 nm (Fig 4). Peak A represented the ultraviolet region type fulvic acid fluorescence peak and peak C represented the class of HA fluorescence peak. In the 3-dimensional map of HA in no year did we observe a fluorescence peak of the protein-like or a fulvic acid-like peak in the visible region were not observed. Studies have shown that the existence of humic acid-like fluorescence peaks represents the complex molecular structure of HA and a high degree of humification, and the fluorescence peaks of fulvic acids are related to the formation of carbonyl and carboxyl groups in the structure of humus [20, 21]. Between the two observed fluorescence peaks, the peak value of the HA peak was larger than the one located in the ultraviolet region. These two characteristic peaks of the relative wavelength were longer, indicating that its molecular weight was relatively high, and that it contains a linear condensed ring and an aromatic ring. Some groups have an unsaturated structure, such as a carbonyl or carboxyl [22, 23]. In addition, the fluorescence index (f 450/500) represents the excitation light wavelength at 370 nm, and the fluorescence intensity ratio at 450 nm and 500 nm in the fluorescence emission spectrum. The f 450/500 value is known to be negatively correlated with HA fragility [24, 25]. A relatively high value of f 450/500 value indicates that the humic substances contain fewer benzene ring structures and are weakly aromatic. In this experiment, the molecular structure of HA was complicated in different fertilization treatments. The f 450/500 values of HA in the CK, NPK, M, and MNPK treatments were 0.583, 0.487, 0.601, 0.618, respectively. It can be inferred that the MNPK treatment has M-treated HA that contains fewer benzene ring structures and that these structures tends to be simplified; the structures of HA in the NPK and CK treatments were more complex.

## Discussion

The application of manure increased the content of SOC and C in HA. The increase in crop yield is associated with the addition of manure or inorganic fertilizer. A single application of manure did not provide enough nutrients for crop growth, whereas the full nutrient supply for crop leads to the increase in crop yield after the application of inorganic N, P and K fertilizer or combined with manure.

Elemental composition analysis is one of the simplest and most important methods used to judge the structure and properties of humus. The proportion of each element in soil HA is usually used to explain trends in its regularity to better analyze the effects of different fertilization treatments. The H/C and O/C ratios generally represents the degrees of condensation and oxidation in soil HA, respectively. That is, a higher H/C ratio indicates a lower degree of condensation, meaning the structure tends to be simple. A larger O/C ratio indicates a higher degree of oxidation. By analyzing elements, we can determine the composition and structure of the chemical content of humus [26]. The H/C ratio is inversely proportional to the degree of condensation of HA and the O/C ratio is proportional to the oxidation degree of HA. There was significant difference in the O/C ratio of HA between different treatments. The application of manure (MNPK and M treatments) increased the ratio of H/C whereas the NPK treatment decreased the ratio of H/C in soil HA, which indicates that soil HA tends to be aliphatic and be simplified after the application of manure, whereas it tends to be complicated after using inorganic fertilizers. Bertoncini [27] and others have shown that the organic C, H, N, and S contents of HA markedly increase in soils amended with sewage sludge compared with in corresponding non-amended soils. Similarly, with higher levels of HA, H/C ratio increase, whereas the O/C ratio decreases [28]. Plaza, et al. (2002) reported that the C, S, and C/N ratios of soil HA increase with the application of pig manure, and this also results in lipidic enhancement [29].

Dou and Hua [30] used the ^13^C-NMR method to study the effects of manure on soil HA and showed that application of organic fertilizer can cause changes in the regularity of carbon skeletons in HA. Generally, application of organic fertilizer results in a decrease in aromatic C and carbonyl C content, but an increase in the content of alkyl C and alkoxy C. These changes lead to fatty acidification of soil HA. Rezaeova et al. [31] showed that the molecular structure of HA and fulvic acid changes after the application of organic fertilizer in that the degree of oxidation degree declines and the number of aromatic structures decreases significantly. In this study, IR analysis of Black Soil treated with different types of fertilizer indicated that NPK and MNPK treatment can both increase the lengths of aliphatic chains in soil HA, so that the ratio of 2920/2720 increased when compared with the CK treatment. The molecular structure of black soil HA becomes aliphatic, simplistic and younger. The CPMAS13C-NMR spectra of black soil HA showed that organic fertilizer treatment could increase the HA content of soil and reduce the aromatic C content, whereas NPK treatment decreased slightly the content of aliphatic C.

Senesi et al. [32] used fluorescence spectroscopy and found that the structure of soil humus affects its fluorescence intensity, and the fluorescence intensity is also affected to some extent by the average molecular weight of soil compounds as well as the source, formation temperature, ionic strength, and oxidation-reduction potential of the humus. When the humus forms a wide and weak peak intensity, it has a higher degree of aromatization with more unsaturated bond and electrophilic functional groups. When the results are reversed, the humus is less humid with fewer unsaturated bonds and fewer electrophilic functional groups, so that the structure is also simple. After the application of manure, the move of the fluorescence wave of HA at 370~450 nm to shortwave indicates a decrease in aromatization degree of soil HA and complicated substituent groups with weak fluorescence intensity (eg. carboxyls and carbonyl), had been substituted with simplified groups with strong fluorescence intensity (eg. hydroxys and amidogens) [33]. Previous results [34, 35] have shown that the fluorescence peak of similar HA indicated the molecule structure of humus could be complicated and humified highly and that of similar fulvic acid was associated with the presence of carbonyl and carboxyl group. Of the two fluorescence peaks observed in the present study, the peak value of the HA peak was larger than that of the ultraviolet region. The f 450/500 values of soil HA in the CK and NPK treatments were lower than those in the MNPK and M treatments, indicating that the degree of aromatization in soil HA was strengthened and that its structure was complicated, whereas treatments with manure added contains fewer benzene ring structures, which means that the structure of the soil tends to be simplified.

## Conclusions

The elemental composition and spectroscopic analyses showed that the application of manure (MNPK and M treatments) increased the contents of element C and aliphatic C in HA, which means the structure of HA was simplified. The application of NPK fertilizer decreased the content of aliphatic C, the ratio of H/C, and the f 450/500 value in HA, all of which indicated that the aromatization degree of soil HA increased and the structure of HA became complicated. Combined applications of these spectroscopic analysis of NMR, IR and fluorescence spectra confirmed these results. In the one rotation cycle, the addition of manure increased the content of aliphatic C, and improved crop yields and soil fertility.
